# Proliferation and apoptosis of T lymphocytes in patients with bipolar disorder

**DOI:** 10.1038/s41598-018-21769-0

**Published:** 2018-02-20

**Authors:** Krzysztof Pietruczuk, Katarzyna A. Lisowska, Karol Grabowski, Jerzy Landowski, Jacek M. Witkowski

**Affiliations:** 1 Department of Physiopathology, Medical University of Gdansk, Gdansk, Poland; 2 Clinic of Adult Psychiatry, Medical University of Gdansk, Gdansk, Poland

## Abstract

The aim of the study was to evaluate proliferation capacity and susceptibility to apoptosis of T lymphocytes of patients with bipolar disorder (BD) and to investigate *in vitro* influence of two standard mood stabilizers: lithium and valproic acid on these parameters using flow cytometry. Our results show that T lymphocytes of BD patients, especially those treated with lithium, have reduced proliferation capacity compared to healthy people. *In vitro* studies showed that valproic acid reduces the number of cell divisions and percentages of proliferating cells regardless of health status but mainly in very high dose, while lithium has no significant influence on proliferation capacity of patients’ T lymphocytes. Lymphocytes of BD patients are also more prone to apoptosis compared with healthy individuals which is related to high expression of Bax, a pro-apoptotic protein. *In vitro* lithium protected patients’ lymphocytes from apoptosis proportionally to dose used. Valproic acid protected lymphocytes of patients from apoptosis mainly in therapeutic concentration. Our results show that mood stabilizers used to prevent relapses of the disease have anti-apoptotic effect on T lymphocytes of BD patients but they are not able to improve their proliferation capacity.

## Introduction

Bipolar disorder (BD) is a serious mental illness with cyclic alternations of mood. It can have a form of depressive and manic episodes in BD type I or depressive and hypomanic episodes in type II but other subtypes and forms of bipolar disorder known as “bipolar spectrum” have been also described^1^. Presently, BD is recognized as a multisystem condition affecting not only patient’s mood and behavior but also endocrine and immune system. In addition to several severe medical conditions accompanying BD, like cardiovascular and metabolic diseases, attention is also being paid to immune dysfunction. This is due to the fact that BD patients are more likely to suffer from cancer^2^ and autoimmune diseases^3^. Moreover, some authors suggest that there is association between immune findings and mood episodes. Thorough investigation of the dysfunction of the immune system in BP is believed to be a key to finding biomarkers that would allow to predict disease progression and even help in treatment selection.

Presently, most of the studies focus mainly on changes in cytokine concentrations in BD. Different authors seem to agree that there is imbalance between pro- and anti-inflammatory cytokines^4–6^. Kim *et al*. have shown that mitogen-induced production of tumor necrosis alpha (TNF-α) and interleukin 6 (IL-6) is significantly higher in bipolar manic patients compared to healthy people^4^. Pandey *et al*. confirmed abnormally high gene expression of pro-inflammatory cytokines and their receptors in lymphocytes isolated from patients with BD^5^. Comparison of cytokine levels in depressed and manic patients revealed that during manic phase there is an increase in the concentration of cytokines such as IL-2, IL-4, IL-6 compared to healthy people, whereas in depressive episode only the level of IL-6 is increased^6^. Breunis *et al*. have demonstrated that bipolar disorder is accompanied by high numbers of circulating activated (CD25^+^) T cells and raised levels of interleukin-2 receptors (sIL-2Rs) compared to healthy control^7^, while Tsai *et al*. showed that increase of sIL-2Rs is higher in patients with acute mania compared to patients in remission^8^. Other authors also demonstrated decrease of interferon gamma (INF-γ) production in acute mania and in subsequent remission compared to healthy people^9^. Changes in the cytokine production could be associated with impaired proliferative responses of T lymphocytes to stimulation, changes in the expression of surface antigens or increased susceptibility to apoptosis.

Treatment of bipolar disorder involves pharmacotherapy and psychotherapy. Types of interventions and drugs used in treatment depend on the type of disorder, its phase and severity. Primary treatment goals are reducing acute symptoms of either depression or hypomania/mania and maintaining stable, euthymic state. Drugs used in treatment of bipolar disorder include antidepressants, antipsychotics and classic mood stabilizers like lithium and antiepileptic agents. Clinicians may choose from drugs more efficient in mania/hypomania (e.g. aripiprazole) or in depression (e.g. lamotrigine) or use more universal agents like lithium. Whereas symptomatic action and clinical effectiveness is well documented and included in various treatment guidelines and recommendations^10,11^, the precise mechanisms of action of different mood stabilizers are not fully understood. But already in the 1980, the effect of lithium on immune cells was demonstrated, when Friedenberg and Marx found that lithium can increase the granulocyte count but reduce their bactericidal capacity^12^. Bray *et al*. a year later showed that the addition of lithium chloride to *in vitro* cultures stimulated mitogenic response of human lymphocytes^13^. More recent work show that lithium can influence gene expression in peripheral lymphocytes; among the genes upregulated during lithium treatment were those related to immune function, like IL5RA and interferon alpha-inducible protein IFI6, and a regulatory factor that affects HLA class II expression (RFX2)^14^. Several studies have shown the effect of lithium on cytokine production by monocytes in breast cancer patients^15–17^. Meanwhile, valproic acid is mainly known for its antileukemic properties; it induces apoptosis of chronic lymphocytic leukemia cells^18,19^.

In the present study we decided to evaluate the efficiency of the immune system of BD patients treated with two different mood stabilizers (lithium or valproic acid). In order to that we analyzed the proliferation capacity of T lymphocytes using modern cytometric technique. To our knowledge, such studies in bipolar disorder do not exist. Since some authors demonstrated that bipolar disorder intensifies apoptosis of patients’ neuronal cells^20^ and peripheral blood lymphocytes^21^, we also examined susceptibility of T lymphocytes of BD patients to apoptosis and analyzed expression of selected proteins involved in process of apoptosis. Additionally, we investigated *in vitro* influence of lithium or valproic acid on these parameters of T lymphocytes in BD patients and healthy people.

## Results

The basic characteristics of BD patients and healthy people participating in the study is given in the Table 1. Briefly, eighteen BD patients in remission, obtaining the maintenance treatment with either lithium or valproic acid, and 10 matched healthy individuals participated, as described in the Methods section below.

**Table 1:** Basic characteristic of BD patients and healthy people.

	Patients (n = 18)	Healthy people (n = 10)
Age (years)	42.87 ± 12.38	43.7 ± 11.35
Sex (M/F)	8/10	4/6
Lithium carbonate (n)	10	—
Valproic acid (n)	8	—
Treatment period (months)	13.52 ± 3.46	—
Beck Depression Inventory	3.62 ± 2.06	2.3 ± 1.41
Hamilton Depression Rating Scale	4.12 ± 1.66	1.9 ± 0.87
Young Mania Rating Scale	2.18 ± 0.65	1.4 ± 0.51

### Comparison of proliferation parameters of T lymphocytes between BD patients and healthy people

**Figure 1:**
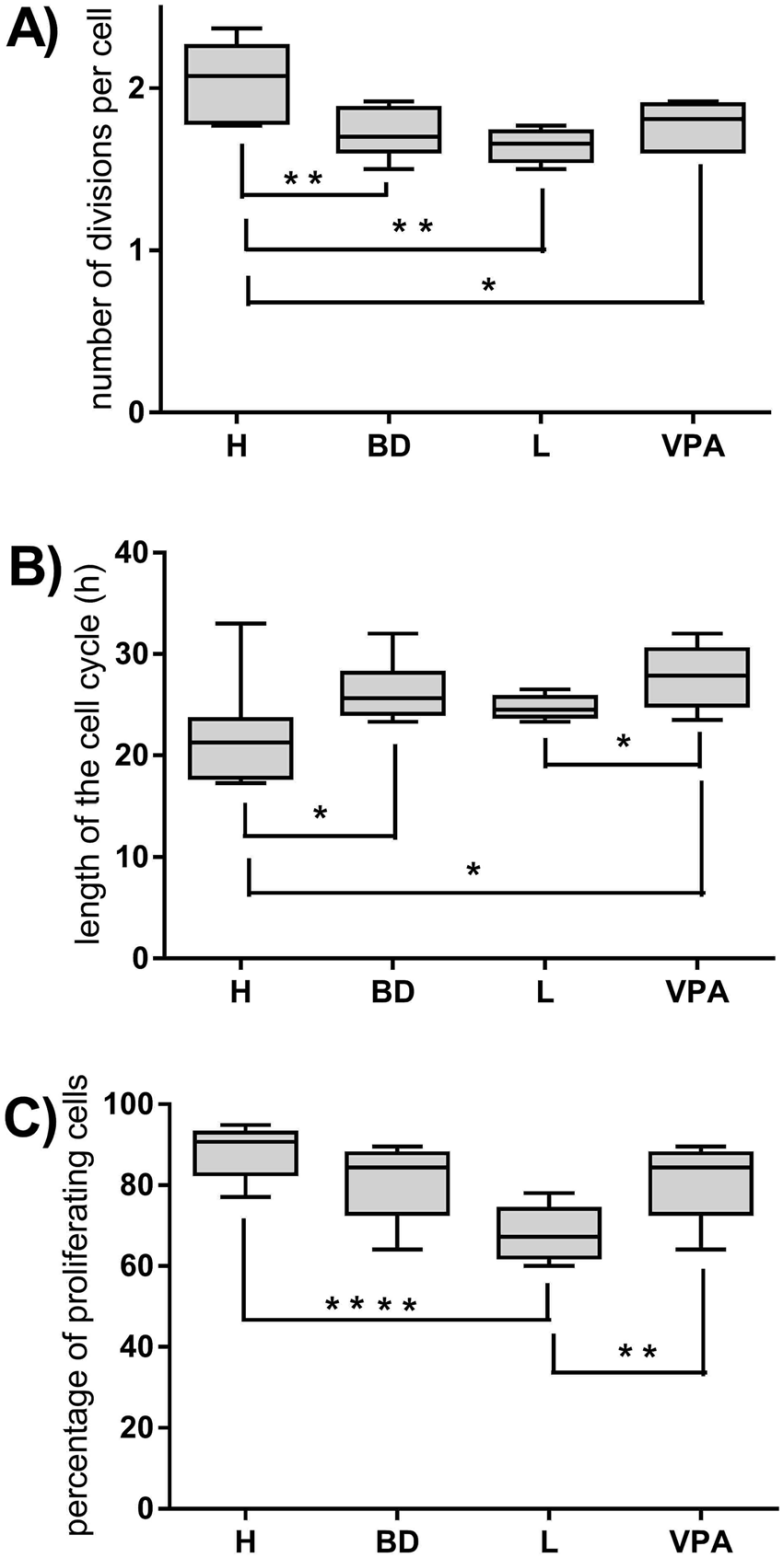
Disease- and treatment-dependent changes in the proliferation parameters of T lymphocytes. Figure **A** shows number of cell divisions per one cell, Figure **B** - length of cell cycle and Figure **C** - percentages of proliferating lymphocytes in healthy people (H), whole group of patients (BD), patients treated with lithium carbonate (L) and patients treated with valproic acid (VPA). Center lines of figures present medians, boxes present 25th-75th percentiles and whiskers show the minimal and maximal values observed, Mann-Whitney test, *p < 0.05, **p < 0.01, ****p < 0.0001.

The proliferation parameters of T lymphocytes stimulated with concanavalin A were compared between healthy people and whole group of BD patients also taking into account the mood stabilizers patients were treated with (lithium or valproic acid). CD4^+^ cells of BD patients performed significantly less divisions per one cell compared to healthy control regardless of type of mood stabilizer used (Fig. 1A). The cell cycle was significantly longer for CD4^+^ cells of BD patients compared to healthy control (Fig. 1B). Moreover, in patients treated with valproic acid lymphocyte time of cell cycle was even longer than in patients treated with lithium. The percentages of proliferating CD4^+^ cells of BD patients treated with lithium were significantly decreased compared to healthy control and patients treated with valproic acid (Fig. 1C).

### *In vitro* influence of lithium and valproic acid on proliferation parameters of T lymphocytes

We also examined the influence of different concentrations of normothymic drugs (lithium or valproic acid) on proliferation parameters of T lymphocytes. Lymphocytes of BD patients treated with lithium were cultured either without or in the presence of lithium, while cells of patients treated with valproic acid were incubated untreated or with valproate. This way we avoided ambiguity in the possible effect of either drug on the cells coming from differently treated patients. Lymphocytes of healthy people were incubated “drug-free” or with either lithium or valproic acid.

**Figure 2:**
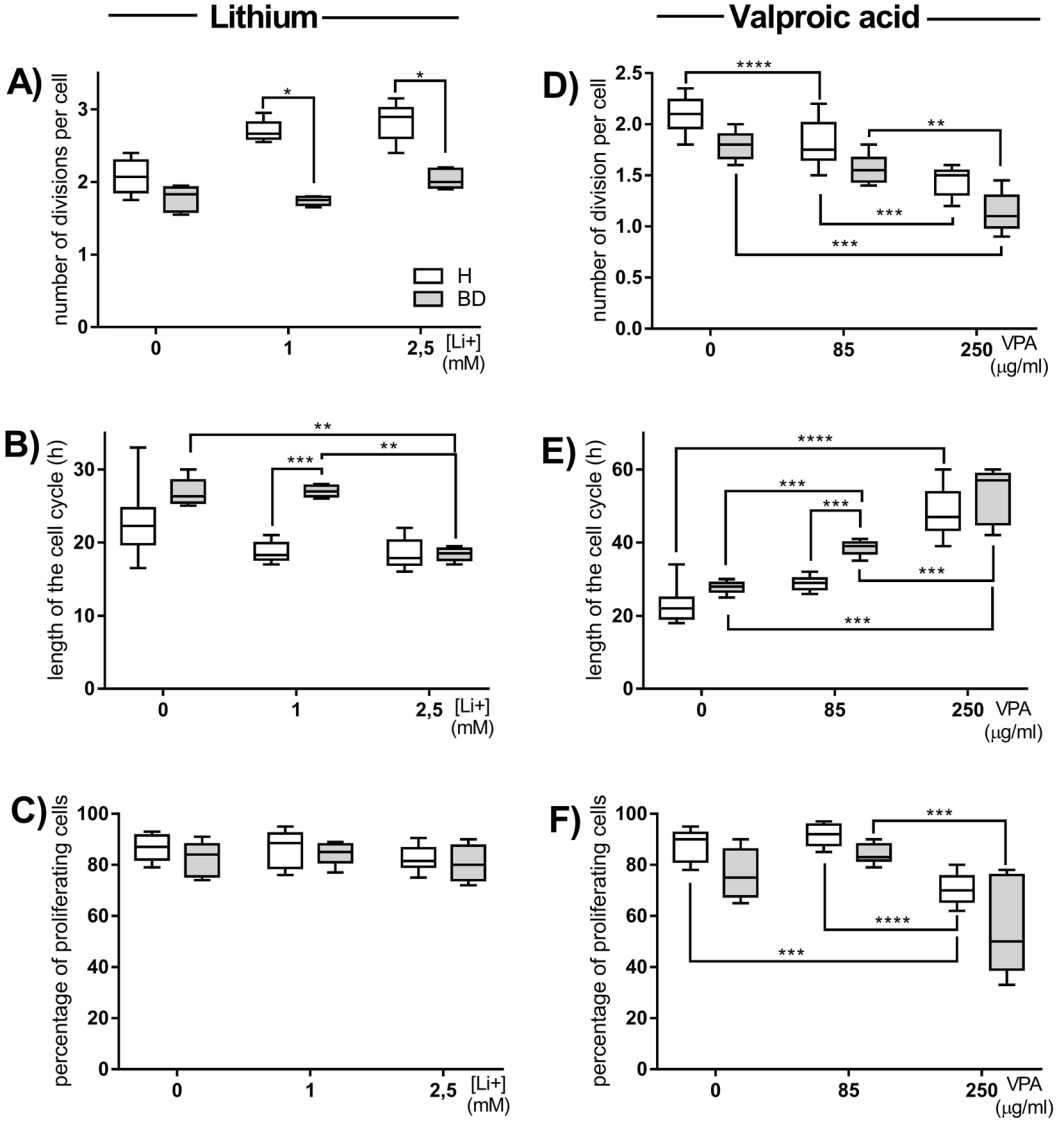
*In vitro* influence of lithium carbonate and valproic acid on the proliferation parameters of T lymphocytes. Figures show cell divisions per one cell (**A**), length of cell cycle (**B**) or percentages of proliferating lymphocytes (**C**) stimulated with 5 µg/ml of concanavalin A alone or in the presence of 1 and 2.5 mM lithium (Li+) in healthy people and BD patients. Figures **D**, **E** and **F** show the same parameters in the presence of 85 and 250 µg/ml valproic acid (VPA). Center lines of figures present medians, boxes present 25th-75th percentiles and whiskers show the minimal and maximal values observed, Mann-Whitney test, Friedman ANOVA and Post Hoc, *p < 0.05, **p < 0.01, ***p < 0.001, ****p < 0.0001.

And so, CD4^+^ cells of healthy people in the presence of 1 or 2.5 mM of lithium performed significantly more divisions per one cell compared to BD patients (Fig. 2A). The cell cycle was significantly shorter for CD4^+^ cells of BD patients but only when 2.5 mM lithium was added (Fig. 2B). No difference in the percentages of proliferating CD4^+^ cells was seen regardless the health status or lithium concentration (Fig. 2C). Meanwhile, in the presence of high (250 µg/ml) concentrations of valproic acid CD4^+^ cells of both healthy people and BD patients performed significantly less divisions per one cell compared with cells stimulated without or in the presence of low (85 µg/ml) concentration of valproic acid (Fig. 2D). The cell cycle was significantly longer for CD4^+^ cells of BD patients in both concentrations of valproic acid (Fig. 2E). The cell cycle was also significantly longer for CD4^+^ cells of healthy people but only in high concentration of valproic acid. The percentage of proliferating CD4^+^ cells of healthy people was significantly decreased but only in the presence of high concentration of valproic acid (Fig. 2F). The percentage of proliferating CD4^+^ cells of BD patients was also significantly decreased in the presence of high concentration of valproic acid but only compared to cells incubated in the presence of low concentration of valproic acid (Fig. 2F).

### Comparison of apoptosis markers of T lymphocytes between BD patients and healthy people

**Figure 3:**
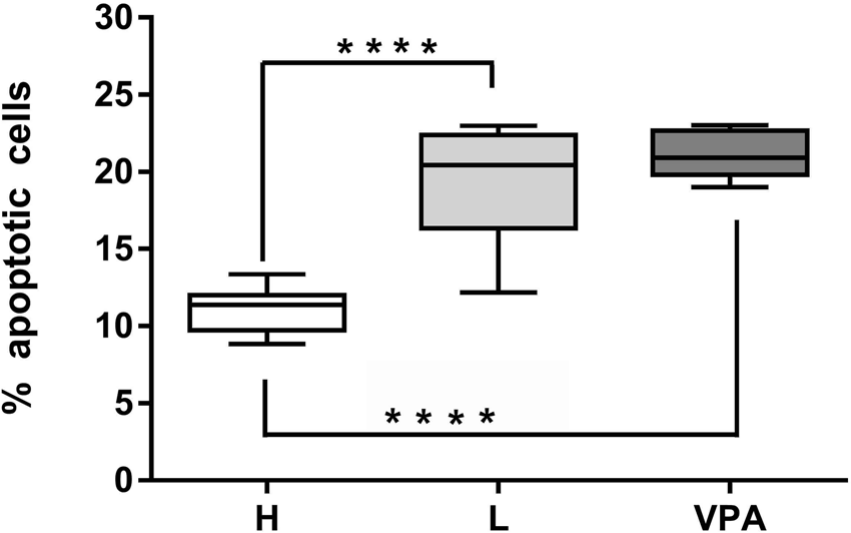
Changes in the percentages of apoptotic T lymphocytes. Figure shows percentage of apoptotic cells compared between healthy control (H) and patients treated either with lithium (L) or valproic acid (VPA). Center lines of figures present medians, boxes present 25th–75th percentiles and whiskers show the minimal and maximal values observed, Mann-Whitney test, ****p < 0.0001.

First, we analyzed activation-induced apoptosis – the type of apoptosis of activated T lymphocytes that results from repeated stimulation of their T-cell receptors (TCR)^22^. We analyzed and compared percentages of apoptotic T cells between BD patients and healthy people taking into account the type of treatment. Percentages of apoptotic lymphocytes were significantly increased in patients treated with lithium or valproic acid compared with healthy control (Fig. 3).

**Figure 4:**
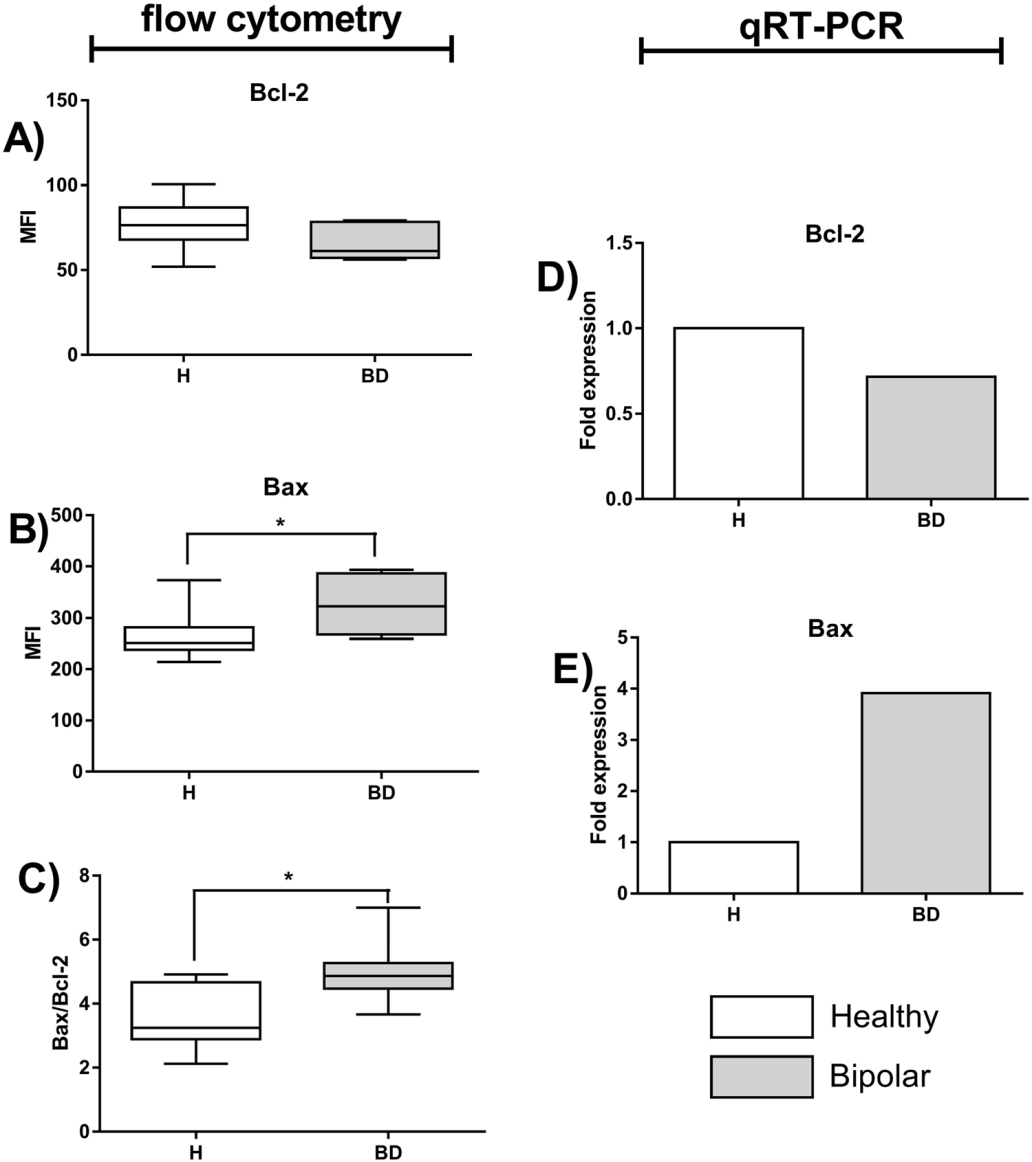
Estimation of Bcl-2 and Bax expression in T lymphocytes. Figure **A** shows expression of Bcl-2 protein measured as mean fluorescence intensity (MFI), Figure **B** - expression of Bax protein and Figure **C** - Bax-Bcl-2 ratio in healthy people and BD patients. Center lines of figures present medians, boxes present 25th-75th percentiles and whiskers show the minimal and maximal values observed, Mann-Whitney test, *p < 0.05. Figure **D** and **E** respectively show fold change in *BCL2* and *BAX* genes’ expression.

We also investigated intracellular level and gene expression of two proteins involved in the process of apoptosis (anti-apoptotic Bcl-2 and pro-apoptotic Bax) in CD4^+^ cells of BD patients and healthy people. We have found no difference in intracellular content of Bcl-2 between examined groups (Fig. 4A). However, CD4^+^ cells of BD patients were characterized by higher expression of Bax compared to healthy people (Fig. 4B), what resulted in increased Bax/Bcl-2 ratio in these cells (Fig. 4C). Molecular study confirmed that expression of *BAX* gene in CD4^+^ cells of BD patients was four times higher compared to healthy control (Fig. 4E).

### *In vitro* influence of lithium and valproic acid on lymphocyte apoptosis in BD patients and healthy people

We have also compared percentages of apoptotic lymphocytes in the presence of agents that activate apoptosis (camptothecin or hydrogen peroxide) as well as normothymic drugs. Lymphocytes of BD patients treated with lithium were cultured in the presence of lithium, while cells of patients treated with valproic acid were incubated with valproate. Lymphocytes of healthy people were incubated with either lithium or valproic acid. Control cultures treated with neither drug were included. The results depended not only on type and concentration of normothymic drug but also on the way apoptosis was induced and healthy status.

**Figure 5:**
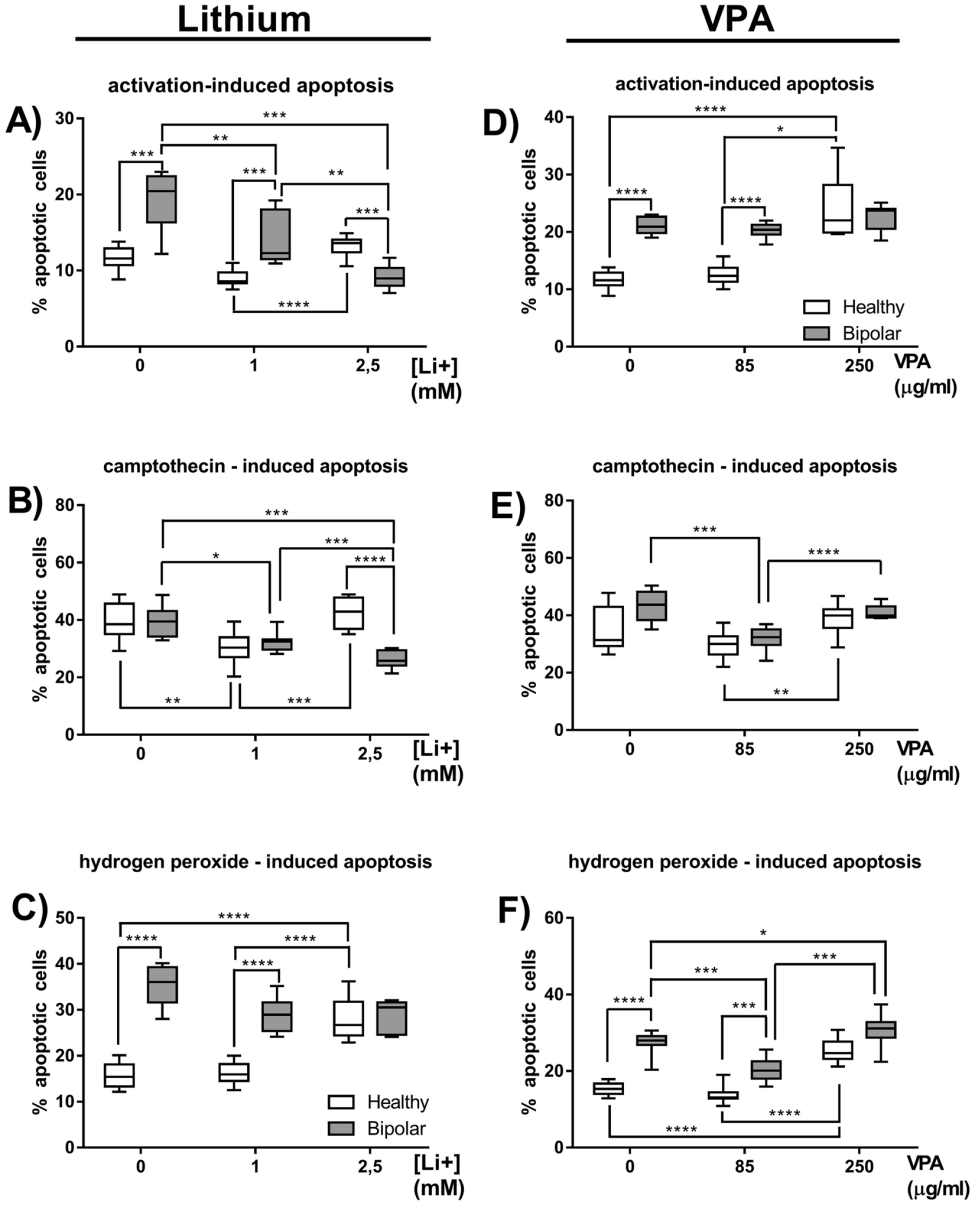
*In vitro* influence of lithium carbonate and valproic acid on the percentages of apoptotic T lymphocytes. Figures show percentages of apoptotic cells as annexin-V-positive cells. Apoptosis of lymphocytes was induced either by activation (through stimulation with concanavalin A) (**A**), camptothecin (**B**) or hydrogen peroxide (**C**) alone or in the presence of 1 or 2.5 mM lithium (Li+) in healthy people and BD patients. Meanwhile Figures (**D**,**E** and **F**) show the same parameters in the presence of 85 or 250 µg/ml valproic acid (VPA). Center lines of figures present medians, boxes present 25th–75th percentiles and whiskers show the minimal and maximal values observed, Mann-Whitney test, Friedman ANOVA and Post Hoc, *p < 0.05, **p < 0.01, ***p < 0.001, ****p < 0.0001.

Percentage of apoptotic cells increased in patients treated with lithium compared to healthy control when apoptosis was induced by activation (Fig. 5A) or hydrogen peroxide (Fig. 5C). *In vitro* treatment with 1 and 2.5 mM of lithium had significantly anti-apoptotic effect on lymphocytes of BD patients when apoptosis was induced by either activation or camptothecin (Fig. 5A and B, respectively). No changes in the percentages of apoptotic cells of BD patients were observed when apoptosis was induced with hydrogen peroxide (Fig. 5C). 1 mM of lithium had no effect on percentage of apoptotic lymphocytes of healthy people regardless the type of apoptosis inducer, while 2.5 mM lithium had significant pro-apoptotic effect on these cells, especially compared with low dose of lithium (Fig. 5A,B and C).

Percentage of apoptotic cells in patients treated with valproic acid was significantly increased compared to healthy control when apoptosis was induced by either activation (Fig. 5D) or hydrogen peroxide (Fig. 5F). Low (85 µg/ml) concentration of valproic acid had no effect on percentage of apoptotic lymphocytes of healthy people regardless the type of apoptosis inducer, while 250 µg/ml of valproic acid had strong pro-apoptotic effect compared to low doses of valproate no matter what type of apoptosis inducer was used (Fig. 5D,E and F) and compared with absence of valproate in cell culture when apoptosis was induced by either activation (Fig. 5D) or hydrogen peroxide (Fig. 5F). Low dose of valproic acid decreased percentages of apoptotic lymphocytes in BD patients when apoptosis was induced by either camptothecin or hydrogen peroxide (Fig. 5E and F, respectively). 250 µg/ml of valproic acid had strong pro-apoptotic effect on patients’ lymphocytes when apoptosis was induced with hydrogen peroxide (Fig. 5F).

## Discussion

Lithium salts and valproic acid are one of the oldest and most commonly used mood stabilizers and they are recommended in maintenance treatment by most treatment guidelines and recommendations^10,11^. At the symptomatic level lithium improves depressive mood, diminishes manic symptoms and prevents suicidality. Valproic acid is used mainly to treat manic episodes or in prophylactic treatment. However, there are a few studies showing that lithium or valproic acid can also influence activity of other cells, especially blood cells. Young *et al*. have noted that lithium often increases white blood cell counts but at the same time reduces blood lymphocyte counts causing lymphopenia^23^. Strangely, although lithium seems to suppress T cell production by inducing thymus involution, at the same time it enhances lymphocytic activity by increasing lymphocyte responses to antigens and mitogens, boosting immunoglobulin synthesis or enhancing natural killer activity^23^. In 1981 Bray *et al*. demonstrated that lithium stimulates mitogenic response of human lymphocytes *in vitro*^13^. In our study we have demonstrated that proliferation capacity of T lymphocytes of BD patients is significantly disturbed compared with healthy people. It concerns especially patients treated with lithium, and to a lesser extent patients treated with valproic acid. Given our *in vitro* results, it cannot be associated with a direct effect of lithium on proliferation parameters of human lymphocytes because regardless of the health status or lithium dose the percentages of proliferating cells stay the same. At the same time the proliferation capacity of lymphocytes of patients treated with valproic acid also remains unchanged in the presence of its therapeutic doses *in vitro*, the number of divisions per cell and percentage of effector cells remains the same even though the length of their cell cycle is longer. Change in the duration of the cell cycle could be related with inhibiting influence of the drug on histone deacetylase (HDAC) activity that leads to arrest in G1/S and G2/M cell cycle check points^24^. This effect becomes evident when patients’ lymphocytes are stimulated in the presence of high dose of valproic acid what results in their decreased proliferation capacity observed as a reduced number of cell divisions and decrease in the percentage of effector cells.

If therapeutic doses of lithium or valproic acid do not interfere with T lymphocyte proliferation in BD patients (as demonstrated *in vitro*), why are the number of effector cells smaller? It could be related to increased susceptibility of patients’ lymphocytes to apoptosis, a process that is essential for limiting inflammation. We have found that percentages of apoptotic lymphocytes were significantly increased in BD patients compared to healthy control regardless the type of mood drug patient was treated with. The direct cause of this phenomenon was the higher expression of Bax protein in patients’ cells. Our result are similar to those obtained by Bei *et al*. who have demonstrated the increase in the level of cytochrome C in cell cytoplasm and Bax translocation leading to increased apoptosis of lymphocytes in the course of BD^25^. Fries *et al*. have also shown that proportions of apoptotic PBMCs are increased in BD patients in remission compared to healthy controls^21^.

The novelty of our study was the use of different methods of inducing cell apoptosis. In general, it appears that lymphocytes of BD patients regardless of mood stabilizer used to treat the disease are more sensitive to activation-induced apoptosis and to apoptosis induced by either DNA damage or oxidative stress. Lymphocytes of healthy people and BD patients react a little bit differently to presence of lihium and valproic acid in cell culture. First of all, both mood stabilizers do not have anti-apoptotic effect on healthy lymphocytes against apoptosis when therapeutic doses are used, while in higher doses they induce apoptosis of healthy lymphocytes. This is different for the patients. Cytoprotective action of lithium is directly proportional to its dose but only when apoptosis is induced by either activation or camptothecin (inhibitor of topoisomerase I). It is not able to protect patients’ lymphocytes from apoptosis induced by oxidative stress. Meanwhile, action of valproic acid depends on dose; in therapeutic concentration it protects patients’ lymphocytes from apoptosis but only when it is induced camptothecin or hydrogen peroxide. In higher doses it has tendency to induce apoptosis of patients’ cells. These results show that anti-apoptotic properties of lithium and valproic acid depend not only on the dose but also health status of cell donors. Lack of reaction of healthy lymphocytes can be related to lack of early contact with either lithium or valproic acid or to lower susceptibility to apoptosis compared with BD patients. In case of valproic acid, increased susceptibility of lymphocytes to apoptosis is probably related to HDAC inhibition, which not only causes cell cycle arrest but also induces apoptosis^24^. Our results confirm that this mood stabilizer not only is safe to use as long as it is administrated in therapeutic doses but also can protect lymphocytes against oxidative stress or other factors affecting DNA synthesis. In turn, molecular mechanism of cytoprotective action of lithium is currently unknown.

According to the psychoneuroimmunology paradigm, there are interactions between mood, behavior and the immune system mediated by endocrine and nervous systems. In healthy people, communication and cooperation between these three system is necessary to maintain homeostasis^26^. Thus, changes in one of these systems reflect and may be in a cause-effect relation with changes observed in the other two, and so analyzing the immune system can be a way to detect changes in nervous system. Since collecting neurons from living people to study cellular and molecular processes is rather difficult, sometimes researchers investigate apoptosis of neurons postmortem and there are studies showing that neurons ofBD patients have an increased expression of pro-apoptotic factors while the expression of anti-apoptotic proteins and synaptic markers is markedly decreased^27^. Our results show that lymphocytes of BD patients are characterized by an increased expression of Bax protein, which disturbs balance between anti- and pro-apoptotic factors. It seems to confirm the theory that changes in nervous system provoke changes in immune system. On the other hand, neurons and immune cells share many common features, e.g. they share molecular mediators of communication and form synapses to communicate with each other. So it is possible that some cellular or molecular changes that lead to neurological disorders in the course of bipolar disorder also affect changes in lymphocyte activity.

An important limitation of the presented results is the small number of examined patients. Despite this, we were able to demonstrate that T lymphocytes of BD patients had significantly reduced proliferation capacity and increased susceptibility to apoptosis compared with healthy people. Lack of untreated patients, however, does not allow us to assess how deep these disturbances are. Even though *in vitro* studies show that therapeutic doses of mood stabilizers used to prevent relapses of the disease have some anti-apoptotic effect on T lymphocytes of BD patients, their susceptibility to apoptosis is still higher compared with healthy people. Therefore, additional studies are necessary to explain cellular and molecular mechanisms of impaired proliferative responses of T lymphocytes.

## Methods

### Patients and healthy controls

The study groups consisted of 18 bipolar disease patients (mean age 42.87 ± 12.38 years) whereas the control group consisted of 10 healthy individuals (mean age 43.7 ± 11.35 years). All participants underwent basic physical examination and psychiatric interview in Clinic of Adult Psychiatry of the Medical University of Gdańsk. Patients were recruited and diagnosed with the Structured Clinical Interview for Diagnostic and Statistical Manual of Mental Disorders Fifth Edition (DSM-5)^28^. The assessment of patients’ mental state was carried out using Beck Depression Inventory^29^, Hamilton Depression Rating Scale^30^ and Young Mania Rating Scale^31^. Only patients who were currently in remission were included in the study. 10 patients were treated with lithium carbonate, 8 with valproate. The dosages of both drugs were similar within groups. The time of therapy with lithium or valproic acid was average 13.05 ± 3.4 months. We excluded people with inflammatory or autoimmunological conditions, diabetes, ischemic heart disease, hypertension and dyslipidemia.

The study was approved by Independent Bioethical Committee for the Scientific Research of the Medical University of Gdańsk. All participants and/or their legal guardians were informed about the purpose of the study and gave their written informed consent. All methods were performed in accordance with the relevant guidelines and regulations.

### PBMC stimulation and dividing cell tracking

20 ml of venous peripheral blood was collected on EDTA from each participant after overnight fasting. Next peripheral blood mononuclear cells (PBMC) were isolated by centrifugation on Histopaque 1077 gradient (Sigma Chemical Co., USA). PBMC were loaded with 1 µM of violet proliferation dye 450 (VPD450, Becton Dickinson) according to the manufacturer’s protocol, distributed to the culture wells, stimulated with 5 µg/ml of concanavalin A (Sigma Chemical Co., USA) alone or in the presence one of normothymic drugs: lithium carbonate (Sigma Chemical Co., USA) or valproic acid sodium salt (Sigma Chemical Co., USA) for 5 days at 37°C, 5% CO_2_. The final concentration of lithium in cell culture was 1 (therapeutic) or 2.5 (toxic) mM^32^, valproic acid – 85 (therapeutic) or 250 (toxic) µg/ml^33^. Cells were collected after 72 and 120 hours, stained with PE-Cy5-conjugated anti-CD4 (Becton Dickinson, USA) and analyzed with flow cytometer.

Proliferation parameters of CD4^+^ lymphocytes were assessed with dividing cell tracking (DCT) method according to our own protocol published earlier^34^ modified by the use of the violet proliferation dye 450 (VPD450) instead of carboxyfluorescein diacetate succinimidyl ester (CFDA-SE).

### Apoptosis assessment

In order to assess apoptosis of activated lymphocytes we used agents that activate apoptosis: 1 µg/ml of camptothecin (inhibitor of topoisomerase I) (Calbiochem, Germany) or 220 μM of hydrogen peroxide (inducer of oxidative stress). First, PBMC were stimulated with concanavalin A, as described above, for 24 hours. Next, camptothecin or hydrogen peroxide alone or in the presence of one of normothymic drugs: lithium carbonate or valproic acid sodium salt were added and cells were incubated for next 72 hours. The final concentration of lithium in cell culture was 1 or 2.5 mM, and of valproic acid −85 or 250 µg/ml. We assessed apoptosis using staining with annexin-V (Annexin-V-FLUOS Staining Kit; Roche, Switzerland).

### Expression of anti-apoptotic and pro-apoptotic proteins

We also investigated intracellular level of anti-apoptotic Bcl-2 and pro-apoptotic Bax in CD4^+^ lymphocytes using intracellular dyes: PE-conjugated anti-Bcl-2 (Becton Dickinson, USA) and Alexa Fluor 647-conjugated anti-Bax (Santa Cruz Biotechnology, USA) antibodies according to manufactures’ protocols.

We also analyzed expression of genes encoding Bcl-2 and Bax in CD4^+^ cells isolated from PBMC using the positive magnetic selection kit - BD IMag™ anti-human CD4 Particles – DM (Becton Dickinson USA). Total RNA from samples containing 3 × 10^6^ of CD4^+^ cells each was isolated using GeneMatrix Universal RNA Purification Kit (EURx, Polska) according to the manufacturer’s protocol. Next, we synthesized complementary DNA (cDNA) using NG dART RT kit (EURx, Polska). Amplification of *BCL2*, *BAX* and *BETA ACTIN* genes was performed with real-time PCR using SensiFAST Probe No-ROX Kit (Bioline USA Inc., USA). The following primers (BLIRT, Poland) were used: 5′-CCTGGGCATGGAGTCCTGT-3′ and 5′-CGTCACACTTCATGATGGAGTTG-3′ for *BETA ACTIN*, 5′-AGATCCGAAAGGAATTGGAATAAA-3′ and 5′-GATTCTGGTGTTTCCCCCTTG-3′ for *BCL2*, 5′-TTTGCTTCAGGGTTTCATCC-3′ and 5′-CAGTTGAAGTTGCCGTCAGA-3′ for *BAX*. The reaction was performed starting with 2 minutes of polymerase activation at 95 °C, followed by 40 cycles composed of 10 seconds of denaturation at 95 °C and 50 seconds of annealing at 60 °C. Thermo Scientific PikoReal 96 cycler and PikoReal Software 2.1 (Thermo Fisher Scientific, USA) were used to analyze the level of *BCL2* and *BAX* expression. Relative expression of the genes was determined with the 2^−ΔΔCT^ method.

### Cytometric analysis

Quantitative cytometric fluorescence analysis was performed with FACSVerse cytometer (Becton Dickinson, USA). At least 10000 lymphocytes (based on their forward and side scatter gating) were acquired from each sample. Cytometric data were analyzed with FlowJo X 10.0.7 (Tree Star; USA). Proliferation parameters were analyzed with numerical protocol described by Witkowski^34^.

### Statistical analysis

Statistical analysis was done with Statistica version 10 (StatSoft Inc., USA). The Kolmogorov-Smirnov and Lilliefors tests were used for testing normality. Since data had non-normal distribution, statistical analysis was done using non-parametric tests: Friedman ANOVA and Post Hoc tests for dependent samples or Mann-Whitney test for independent samples with the level of significance p < 0.05.
